# Modeling the evolution of *Schizosaccharomyces pombe* populations with multiple killer meiotic drivers

**DOI:** 10.1093/g3journal/jkae142

**Published:** 2024-06-28

**Authors:** José Fabricio López Hernández, Boris Y Rubinstein, Robert L Unckless, Sarah E Zanders

**Affiliations:** Stowers Institute for Medical Research, 1000 East 50th Street, Kansas City, MO 64110, USA; Stowers Institute for Medical Research, 1000 East 50th Street, Kansas City, MO 64110, USA; Department of Molecular Biosciences, University of Kansas, 1200 Sunnyside Avenue, Lawrence, KS 66045, USA; Stowers Institute for Medical Research, 1000 East 50th Street, Kansas City, MO 64110, USA; Department of Cell Biology and Physiology, University of Kansas Medical Center, 3901 Rainbow Boulevard, Kansas City, KS 66160, USA

**Keywords:** meiotic driver, gamete killers, gene drive, 2-locus evolution

## Abstract

Meiotic drivers are selfish genetic loci that can be transmitted to more than half of the viable gametes produced by a heterozygote. This biased transmission gives meiotic drivers an evolutionary advantage that can allow them to spread over generations until all members of a population carry the driver. This evolutionary power can also be exploited to modify natural populations using synthetic drivers known as “gene drives.” Recently, it has become clear that natural drivers can spread within genomes to birth multicopy gene families. To understand intragenomic spread of drivers, we model the evolution of 2 or more distinct meiotic drivers in a population. We employ the *wtf* killer meiotic drivers from *Schizosaccharomyces pombe*, which are multicopy in all sequenced isolates, as models. We find that a duplicate *wtf* driver identical to the parent gene can spread in a population unless, or until, the original driver is fixed. When the duplicate driver diverges to be distinct from the parent gene, we find that both drivers spread to fixation under most conditions, but both drivers can be lost under some conditions. Finally, we show that stronger drivers make weaker drivers go extinct in most, but not all, polymorphic populations with absolutely linked drivers. These results reveal the strong potential for natural meiotic drive loci to duplicate and diverge within genomes. Our findings also highlight duplication potential as a factor to consider in the design of synthetic gene drives.

## Introduction

Most alleles are Mendelian in that they are transmitted to half of the offspring of a given individual. Meiotic drive alleles, in contrast, can be passed on to more than half, even all offspring. Meiotic drive is a powerful evolutionary force as the transmission bias allows a meiotic driver to spread in a population (Sandler and Novitski 1957). Understanding the spread of meiotic drivers within populations is critical for deciphering the evolution of natural populations and may guide design of synthetic gene drives that aim to control natural populations (Lindholm *et al*. 2016; Zanders and Unckless 2019; Price *et al*. 2020).

The evolution of single drive loci in populations has been extensively modeled (Hartl 1970; Crow 1991; Fishman and Kelly 2015; Bull 2016; Hall and Dawe 2018; Dyer and Hall 2019; Manser *et al*. 2020; López Hernández *et al*. 2021; Martinossi-Allibert *et al*. 2021). However, some species carry multiple, unrelated meiotic drivers (Voelker and Kojima 1971; Cazemajor *et al*. 2000; Dalstra *et al*. 2003; Lyon 2003; Tao *et al*. 2007; Long *et al*. 2008; Yang *et al*. 2012; Didion *et al*. 2015; Yu *et al*. 2018; Akera *et al*. 2019; Vogan *et al*. 2019; Bravo Núñez, Sabbarini, Eickbush, *et al*. 2020). Additionally, some drive genes are members of multigene families (Hu *et al*. 2017; Nuckolls *et al*. 2017; Dawe *et al*. 2018; Vogan *et al* 2019; Muirhead and Presgraves 2021; Vedanayagam *et al*. 2021). One potential evolutionary implication of species carrying multiple distinct allelic drivers, namely, selection for reduced fidelity of meiosis, has recently been explored using evolutionary modeling (Bravo Núñez, Sabbarini, Eide, *et al*. 2020). However, the evolution of populations polymorphic for multiple drivers born from gene duplication has not been formally considered.

**Fig. 1.**
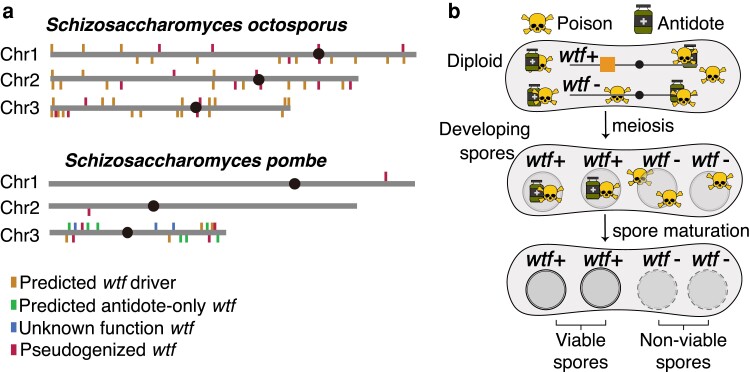
Poison–antidote *wtf* meiotic drivers in *Schizosaccharomyces*. a) Genomic loci that contain members of the *wtf* gene family in *Schizosaccharomyces octosporus* and *S. pombe* reference genomes (Eickbush *et al*. 2019; De Carvalho *et al*. 2022). Each marked locus contains at least one of the indicated *wtf* genes. b) In *S. pombe*, a *wtf* meiotic driver produces both a poison and an antidote that are expressed in diploids induced to undergo meiosis. After meiosis, the antidote is enriched only in the spores that inherit the driver. The antidote rescues only the cells that inherit the driver, while the rest of the spores are susceptible to the poison.

The *wtf* killer meiotic drivers found in fission yeasts (*Schizosaccharomycetes*) have undergone many gene duplication events over the past ∼119 million years (Fig. 1a; De Carvalho *et al*. 2022). In *Schizosaccharomyces pombe*, distinct isolates encode between 4 and 14 genes that appear to be intact drivers (Hu *et al*. 2017; Eickbush *et al*. 2019). Each *wtf* driver encodes a poison and an antidote protein from separate, but largely overlapping, transcripts of the same gene. All 4 developing meiotic products (spores) are exposed to the poison, while only those that inherit the driving *wtf* gene acquire enough antidote to neutralize the poison (Fig. 1b; Hu *et al*. 2017; Nuckolls *et al*. 2017; Nuckolls *et al*. 2022). Importantly, the antidotes encoded by a given *wtf* driver generally provide no protection against the poisons of distinct drivers with different sequences (Hu *et al*. 2017; Bravo Núñez, Sabbarini, Eide, *et al*. 2020).

Most of the characterized *wtf* drive genes show strong transmission (>80%) from heterozygotes, particularly in *S. pombe*, the species where the genes have been studied the most (Hu *et al*. 2017; Bravo Núñez, Sabbarini, Eickbush, *et al*. 2020). In addition, the *wtf* drivers also often act unopposed by suppressors, although suppressors do exist (Bravo Núñez *et al*. 2018). From a classic population genetics viewpoint, these factors suggest that the *wtf* drivers would rapidly spread to fixation a population. This rapid fixation is observed for single drivers in laboratory populations, but surprisingly, this prediction of driver fixation is not borne out by allele frequencies observed in *S. pombe* (Eickbush *et al*. 2019; López Hernández *et al*. 2021).

Instead of sharing fixed drivers, the *wtf* genes in *S. pombe* are strikingly polymorphic. Distinct isolates of *S. pombe* have different numbers of *wtf* drivers, ranging from 4 to 14 and, none of them are fixed in the species. At a given locus, 2 *S. pombe* isolates may both encode a *wtf* diver, but the sequences tend to be different and can thus be potentially distinct (mutually killing) due to extremely rapid evolution (Eickbush *et al*. 2019). For example, 2 of the most intensively studied *S. pombe* isolates both encode a driver at the *wtf4* locus, but they are mutually killing (Bravo Núñez, Sabbarini, Eide, *et al*. 2020). Moreover, the patterns of rapid *wtf* gene evolution found in *S. pombe* are shared with other fission yeast species. This suggests an ongoing cycle of driver birth, rapid divergence, and potentially sustained polymorphism over the past ∼119 million years (De Carvalho *et al*. 2022).

To better understand the evolution of the *wtf* drivers, and perhaps other drive gene families, we reasoned models must consider more than 1 segregating drive loci. As a first step toward achieving this goal, we modeled the evolution of 2 *wtf* meiotic drive loci. We found that both *wtf* drivers are likely to spread in a population under many conditions, particularly when the genes diverge to become distinct drivers. Overall, our results help explain both the duplication *wtf* drivers into a gene family and the selective incentive for *wtf* gene divergence after duplication, even in the absence of suppressors.

## Materials and methods

### Model for identical *wtf* drivers

*S. pombe* cells generally grow asexually as haploids when resources are abundant. This means populations can be founded by 1 or more haploid genotypes that can clonally expand without sexual reproduction. When starved, haploid *S. pombe* cells can mate to form a diploid that undergoes meiosis to produce 4 haploid progenies, known as spores (Forsburg and Rhind 2006). While the relative time spent in the haploid phase is different from diploid eukaryotes, the same types of equations can be used to model allele frequency changes over generations of sexual reproduction (Crow 1991; López Hernández *et al*. 2021).

We initially modeled the evolution of a pair of identical *wtf* driver duplicates, *wtfA* and *wtfB*, at distinct loci over successive rounds of sexual reproduction. Our equations are extensions of those presented in Crow (1991). Each driver has only 1 alternate allele that does not drive (e.g. *wtfA*−). A total of 4 distinct haploid genotypes are therefore possible: *wtfA*+ *wtfB*+, *wtfA*+ *wtfB*−, *wtfA*− *wtfB*+, and *wtfA*− *wtfB*−. Those genotypes are found with frequencies $x_{1}$, $x_{2}$, $x_{3}$, and $x_{4}$, respectively (Table 1). We assume an infinitely large population, equal fitness of all haploid genotypes during clonal growth, and random mating. While some of the genotype compositions we model would be atypical for unlinked genes in exclusively sexually reproducing organisms at Hardy–Weinberg equilibrium, they are reasonable for organisms like *S. pombe* that do both asexual and sexual reproductions (López Hernández *et al*. 2021). For example, one could have an *S. pombe* population that has equal numbers of *wtfA*+ *wtfB*+ and *wtfA*− *wtfB*− individuals at the time of sexual reproduction, even if *wtfA* and *wtfB* loci are unlinked.

**Table 1.** Parameters and variables used in the modeling of 2 drivers in a fission yeast population.

Parameters/variables	Description	Parameter range
$x_{1}$	Frequency of genotype *wtfA*+ *wtfB*+	0–1
$x_{2}$	Frequency of genotype *wtfA*+ *wtfB*−	0–1
$x_{3}$	Frequency of genotype *wtfA*− *wtfB*+	0–1
$x_{4}$	Frequency of genotype *wtfA*− *wtfB*−	0–1
*t*	Transmission advantage	0–1
*r*	Recombination frequency between *wtf* loci	0–0.5

**Fig. 2.**
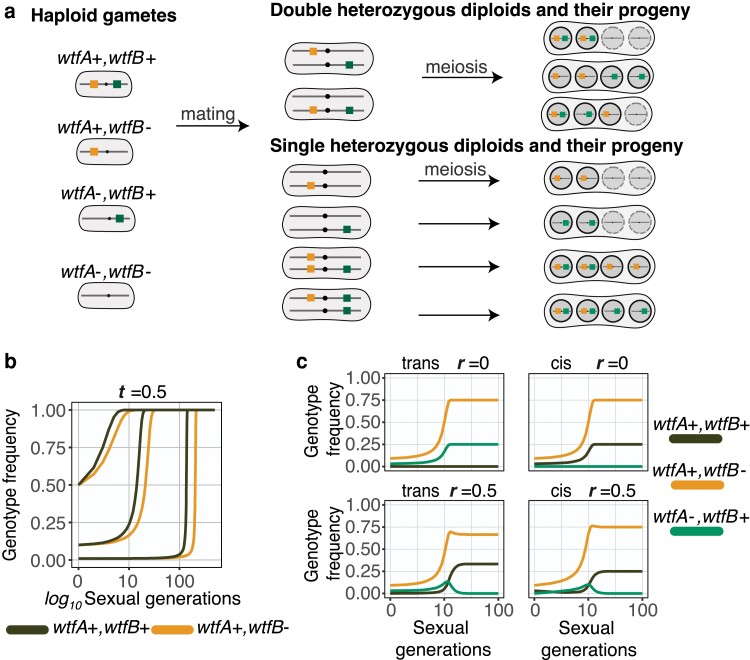
Evolution of 2 identical drivers after gene duplication. a) Four distinct genotypes are possible in the population after a *wtf* driver (*wtfA+*) duplicates: *wtfA*+ *wtfB*+, *wtfA*+ *wtfB*−, *wtfA*− *wtfB*+, and *wtfA*− *wtfB*−. Those haploids can mate to form diploids with a variety of genotypes. Drive will occur if diploids are heterozygous for 1 or 2 drivers. Spores that do not inherit one of the drivers from a heterozygote are susceptible to killing. Live spores are shown within a solid black circle whereas spores susceptible to killing by drive are shown within a dotted circle. b) Simulations of genotypes with 1 driver (*wtfA*+ *wtfB*−, orange) or 2 drivers absolutely linked in cis (*wtfA*+ *wtfB*+, black) spreading in a population where the alternate genotype lacks drivers (*wtfA*− *wtfB*−). The initial frequencies of the *wtfA*+ *wtfB*− and *wtfA*+ *wtfB*+ genotypes shown are 0.01, 0.1, and 0.5. The transmission advantage (*t*) for each driver is 0.5. c) Four distinct simulations in which a driver (*wtfA*+) makes an identical duplicate (*wtfB+*) in trans (on the homologous chromosome, left) or in cis (on the same chromosome, right). The transmission advantage (*t*) for each driver is 1. Simulations where the duplicate gene is absolutely linked (*r* = 0, top) and unlinked (*r* = 0.5, bottom) from the parent gene are shown. The starting frequency of the ancestral genotype (*wtfA*+ *wtfB*−, orange) is 0.1. The starting frequency of genotypes with a duplicated driver in cis (*wtfA*+ *wtfB*+, black) or in trans (*wtfA*− *wtfB*+, green) is 0.03. The remainder of each population is comprised of the *wtfA*− *wtfB*− genotype.

As the drivers are identical, drive (spore killing) will only affect spores that inherit no *wtf*+ alleles from a diploid carrying 1 or more *wtf*+ alleles (Fig. 2a). The parameter “*t*” is the fraction of spores not inheriting the drivers that are killed, so it thus represents the strength of drive (Table 1). Spores that inherit neither *wtfA*+ or *wtfB*+ from a diploid cell heterozygous for both are susceptible to killing by both drivers (i.e. a fraction represented by $2t-t^{2}$ are killed and $(1-t{)}^{2}$ survive since $t_{A}=t_{b}=t$). We assign no additional fitness costs to any genotypes, beyond the costs caused by the driver due to spore killing.

The frequency at which recombinant genotypes form in the spore of double heterozygotes (e.g. *wtfA*+ *wtfB*+/*wtfA*− *wtfB*−) diploids is determined by “*r*” (Table 1). To simplify calculating genotype frequency changes due to recombination during gametogenesis, we use the parameter “*E*” where $E=r(x_{1}x_{4}-x_{2}x_{3})$ (equal to “D” in Crow 1991). The frequency of each genotype in subsequent generations of a given starting population can be calculated using Equations 1.1–1.4. Each equation includes a parameter for mean population fitness ($\bar{w})$, which is defined in Equation 1.5. To calculate the frequency of *wtfA*+ *wtfB*+ spores in the next generation ($x_{1}^{\prime }$), we considered all possible diploid genotypes that can generate *wtfA*+ *wtfB*+ spores:


$$x_{1}^{\prime }=\frac{1}{\bar{w}}(x_{1}^{2}+x_{1}x_{2}+x_{1}x_{3}+x_{1}x_{4}-E).$$

After considering that $x_{1}+x_{2}+x_{3}+x_{4}=1$, we can further simplify to


(1.1) $$x_{1}^{\prime }=\frac{1}{\bar{w}}(x_{1}-E).$$

To calculate the frequency of the *wtfA*+ *wtfB*− and *wtfA*− *wtfB*+ genotypes in the next generation ($x_{2}^{\prime }$ and $x_{3}^{\prime }$, respectively), we can use very similar equations as that used to calculate $x_{1}^{\prime }$; however, we use “+E” to reflect the change in the 2 genotypes due to recombination as follows:


(1.2) $$x_{2}^{\prime }=\frac{1}{\bar{w}}(x_{2}+E)\;\mathrm{and}$$

(1.3) $$x_{3}^{\prime }=\frac{1}{\bar{w}}(x_{3}+E).$$

To calculate the frequency of spores with the *wtfA*− *wtfB*− genotype in the next generation ($x_{4}^{\prime }$), we must consider that those spores are susceptible to killing. When *wtfA*− *wtfB*− spores are generated by a single heterozygote, (1−t) spores survive, whereas $(1-t{)}^{2}$  *wtfA*− *wtfB*− spores survive when they are generated by a diploid heterozygous for both drivers. Considering the fitness of each diploid, we can calculate $x_{4}^{,}$ with the equation below.


$$x_{4}^{\prime }=\frac{1}{\bar{w}}(x_{4}^{2}+x_{2}x_{4}(1-t)+x_{3}x_{4}(1-t)+(x_{1}x_{4}-E){\left(1-t\right)}^{2}),$$

and given that $x_{1}+x_{2}+x_{3}+x_{4}=1$, the equation can be simplified to:


(1.4) $$x_{4}^{\prime }=\frac{1}{\bar{w}}(x_{4}(1-t(2x_{1}+x_{2}+x_{3})+t^{2}x_{1})-E{\left(1-t\right)}^{2}).$$

To calculate the mean population fitness ($\bar{w}$), we used the surviving spores produced by all genotypes and their frequencies. The fitness of the $x_{1}$, $x_{2}$, and $x_{3}$ genotypes is 1. For $x_{4}$, a fraction of spores are destroyed by drive. The derivation of the fitness of $x_{4}$ spores is taken from Equation 1.4.


$$\bar{w}=\;x_{1}-E+x_{2}+E+x_{3}+E+x_{4}(1-t(2x_{1}+x_{2}+x_{3})+t^{2}x_{1})-E(1-t{)}^{2}.$$

Given that $x_{1}+x_{2}+x_{3}+x_{4}=1$, the equation can be simplified to:


(1.5) $$\bar{w}=\;1+x_{4}t(-(2x_{1}+x_{2}+x_{3})+tx_{1})+2Et(1-t).$$

### Model for distinct *wtf* drivers

The model for 2 distinct *wtf* drivers (again represented by *wtfA*+ and *wtfB*+) uses the same parameters and is similar to the model for identical *wtf* drivers (described above) with 1 important difference. Namely, spores that inherit *wtfA+* are not protected from killing by *wtfB+* and vice versa. Thus, if a diploid is heterozygous for both drivers, a spore must inherit both to be resistant to killing. Because of this, the fitness components of the equations to calculate $x_{2}^{\prime }$ through $\;x_{4}^{\prime }$ change is described below.

To calculate the frequency of *wtfA*+ *wtfB*− spores in the next generation ($x_{2}^{\prime }$), we calculated that the *wtfB*+ driver will kill a fraction of *wtfA*+ *wtfB*− spores (described by $t_{B}$) generated by diploids heterozygous for *wtfB* as shown below.


(2.1) $$x_{2}^{\prime }=\frac{1}{\bar{w}}(x_{2}^{2}+x_{1}x_{2}(1-t_{B})+x_{2}x_{4}+(x_{2}x_{3}+E)(1-t_{B})).$$

This can be simplified to:


(2.2) $$x_{2}^{,}=\frac{1}{\bar{w}}(x_{2}(1-t_{B}(x_{1}+x_{3}))+E(1-t_{B})).$$

The equation for calculating the frequency of *wtfA*− *wtfB*+ spores in the next generation ($x_{3}^{\prime }$), must be similarly amended to include that the *wtfA*+ driver will kill a fraction of *wtfA*− *wtfB+* spores (described by $t_{A}$) generated by diploids heterozygous for *wtfA* as shown below.


$$x_{3}^{\prime }=\frac{1}{\bar{w}}(x_{3}^{2}+x_{1}x_{3}(1-t_{A})+x_{3}x_{4}+(x_{2}x_{3}+E)(1-t_{A})).$$

This can be simplified to:


(2.3) $$x_{3}^{\prime }=\frac{1}{\bar{w}}(x_{3}(1-t_{A}(x_{1}+x_{2}))+E(1-t_{A})).$$

To calculate the frequency of *wtfA*− *wtfB*− spores in the next generation ($x_{4}^{\prime }$), we modified the equation to reflect that these spores are sensitive to being killed by *wtfA+* in *wtfA*+ *wtfB*−/*wtfA*− *wtfB*− diploids, by *wtfB*+ in *wtfA*− *wtfB*−/*wtfA*− *wtfB*+ diploids, and by both drivers in diploids heterozygous for both drivers as shown below.


$$x_{4}^{\prime }=\frac{1}{\bar{w}}(x_{4}^{2}+x_{2}x_{4}(1-t_{A})+x_{3}x_{4}(1-t_{B})+(x_{1}x_{4}-E)(1-t_{A})(1-t_{B})).$$

This can be simplified to:


(2.4) $$\begin{array}{r} x_{4}^{\prime }=\frac{1}{\bar{w}}(x_{4}(1-t_{A}(x_{1}+x_{2})-t_{B}(x_{1}+x_{3})+x_{1}t_{A}t_{B})-E(1-t_{A})(1-t_{B})) \end{array}.$$

The mean population fitness is again calculated by considering the fitness of all genotypes in the population as follows:


$$\begin{array}{rl} \bar{w} & =x_{1}-E+x_{2}(1-t_{B}(x_{1}+x_{3}))+E(1-t_{B})+x_{3}(1-t_{A}(x_{1}+x_{2}))\\ & \;+E(1-t_{A})+x_{4}(1-t_{A}(x_{1}+x_{2})-t_{B}(x_{1}+x_{3})+x_{1}t_{A}t_{B})\\ & \;-E(1-t_{A})(1-t_{B}). \end{array}$$

This can be simplified to:


(2.5) $$\begin{array}{r} \bar{w}=1-(x_{2}+x_{4})(x_{1}+x_{3})t_{B}-(x_{3}+x_{4})(x_{1}+x_{2})t_{A}+(x_{1}x_{4}-E)t_{A}t_{B} \end{array}.$$

### Model for 2 distinct drivers on competing haplotypes

To model the evolution of 2 distinct driver alleles at a single locus, we assumed no recombination between drivers (r=0). We designated 2 possible driver alleles: *wtfA^1^* and *wtfA^2^*, with the relative frequencies $x_{A^{1}}^{\prime }$ and $x_{A^{2}}^{\prime }$, respectively. The spore killing caused by each driver is defined by the *t* value for that driver. Drive will occur in heterozygotes such that each spore is susceptible to being killed by the driver it does not inherit. Drive does not, however, occur in homozygotes.

The frequency of each allele in subsequent generations can be calculated as follows:


(3.1) $$x_{A^{1}}^{\prime }=\frac{1}{\bar{w}}(x_{A^{1}}(1-x_{A^{2}}t_{A^{2}})),$$

(3.2) $$x_{A^{2}}^{\prime }=\frac{1}{\bar{w}}(x_{A^{2}}(1-x_{A^{1}}t_{A^{1}})),$$

where mean population fitness was


(3.3) $$\bar{w}=1-x_{A^{1}}x_{A^{2}}(t_{A^{1}}+t_{A^{2}}).$$

### Steady-state solutions and stability analysis

We determined possible genotype frequency steady-state solutions using Mathematica (Wolfram Research 2021). We defined the steady state of the recurrence equations by identifying that the equations follow the form: $x_{i}\bar{w}=f(x_{i})$. Here, x_i_^′^ is the frequency of each genotype “*i*” to the next generation which depends on the mean population fitness $\bar{w}$ and a function of the absolute frequency of each genotype $f(x_{i})$. The steady state is determined by the condition in which the change of all genotypes to the next generation equals 0: $x_{i}\bar{w}-f(x_{i})=0$.

Steady-state solutions were determined by simplifying the system of equations to $x_{4}=1-x_{1}-x_{2}-x_{3}$. Solutions were found for the cases r=0 or $t_{A}=t_{B}$ including a particular case where $t_{A}=t_{B}=1$. When 2 competing haplotype drivers are present, $x_{A^{2}}=1-x_{A^{1}}$.

To determine the mathematical stability of the solutions to small perturbations, we used the eigenvalues of the Jacobian matrix for all 4 recurrence equations (Otto and Day 2007). A solution is stable only when the leading eigenvalue is less than 1 and unstable when it is greater than 1. In cases where the associated eigenvalues are exactly 1, the solution stability cannot be defined by the Jacobian matrix alone. The solution when the *wtfA*+ *wtfB*+ genotype is fixed is not defined by the Jacobian except upon perturbation of the genotype frequencies (Table 2; see Supplementary material for mathematical proof).

**Table 2.** The solutions and stability associated to leading eigenvalues for 2 distinct drivers (see Supplementary material for complete description).

Class	Conditions			Stability
	r	t	Genotype frequencies	
I	0≤r≤0.5	$0<t_{A},t_{B}<1$	$x_{1}=1,x_{2}=0,x_{3}=0,x_{4}=0$	Stable
			$x_{1}=0,x_{2}=1,x_{3}=0,x_{4}=0$ , $x_{1}=0,x_{2}=0,x_{3}=1,x_{4}=0$, $x_{1}=0,x_{2}=0,x_{3}=0,x_{4}=1$.	Unstable
II	0<r≤0.5	t=1	$x_{1}=\frac{r}{1+r},x_{2}=0,x_{3}=0,x_{4}=\frac{1}{1+r}$	Unstable
III	r=0	$0<t_{A^{1}}\leq 1,$ $0<t_{A^{2}}\leq 1$	$x_{A^{1}}=\frac{t_{A^{2}}}{t_{A^{1}}+t_{A^{2}}},x_{A^{2}}=\frac{t_{A^{1}}}{t_{A^{1}}+t_{A^{2}}}$	Unstable

### Simulations, data analysis, and visualization

We carried out deterministic forward simulations, using a range of starting genotype frequencies, to describe the evolution of genotypes that lead to the found steady-state solutions. For all simulations, we assumed an infinitely large population and simulated 10,000 generations or until a steady state with a genotype frequency change less than 1 * 10^−15^ occurred. For each generation, we tracked and updated the genotype frequencies and mean population fitness. To determine the fate of each genotype, all frequencies close to 1 or 0 were rounded with tolerance of 1 * 10^−13^, lower than the inverse reported effective population size 1 * 10^5^–1 * 10^9^ (Farlow *et al*. 2015; Tusso *et al*. 2019). A genotype was considered fixed when it equaled 1 and extinct when it equaled 0.

All simulations were coded in and performed using R (Team 2019. Version 4.2.3) with the packages ggplot (Wickham 2016), ggtern (Hamilton and Ferry 2018), and viridis (Garnier et al. 2024). The code is available at https://github.com/Zanders-Lab/Modeling_the_evolution_of_populations_with_multiple_killer_meiotic_drivers.

## Results

### Evolution of 2 identical *wtf* paralogs

Initially after a gene duplication, the 2 meiotic driver paralogs are likely to be identical. We thus first considered the evolution of 2 identical drivers: *wtfA* and its paralog *wtfB*. We considered gene duplication events that were absolutely linked in cis (e.g. a tandem duplication) and absolutely linked in trans (e.g. a duplication to the competing haplotype, which could occur in the diploid phase). We also considered duplications to a locus unlinked to *wtfA*.

Briefly, our model considers an infinitely large population, random mating, and no fitness costs beyond the fraction of spores destroyed by drive. There are 4 haploid genotypes possible: *wtfA*+ *wtfB*+, *wtfA*+ *wtfB−*, *wtfA− wtfB*+, and *wtfA− wtfB−*. Because *wtfA* and *wtfB* are identical, drive will occur in diploids that are (1) heterozygous for both drivers and (2) in diploids heterozygous for 1 driver and lacking the second driver. In both cases, only spores that that do not inherit either driver (*wtfA− wtfB−*) can be destroyed by drive (Fig. 2a). We use the term “*t*” to reflect the transmission advantage of each driver in heterozygotes. For example, at t=1, all *wtfA− wtfB−* spores produced by diploids heterozygous for both drivers would be destroyed. At t=0.5, 75% $\left(2t-t^{2}\right)$ of the *wtfA− wtfB−* spores from diploids heterozygous for both drivers are destroyed. We used the parameter “*r*” to reflect recombination frequencies. We modeled populations with varying starting frequencies of the 4 haploid genotypes.

With a tandem identical *wtf* gene duplicate (i.e. *wtfA*+ *wtfB*+ absolutely linked in cis; r=0), we found that the *wtfA*+ *wtfB*+ could spread in a population of *wtfA*− *wtfB*− cells faster than a haplotype containing a single driver locus (*wtfA*+ *wtfB−*) when t<1 (Fig. 2b). The rate of spread of a single drive gene asymptotically approaches the rate of spread of 2 identical tandem drivers as *t* approaches 1. If a driver with the strongest possible transmission advantage (t=1) makes a tandem duplicate, the dynamics of driver spread are the same as if the duplicate did not occur. After fixation of *wtfA*+, drive no longer occurs and allele frequencies remain constant due to the “immunity” gained by the presence of *wtfA*+ (Fig. 2c).

In the less likely, but possible, scenario that the identical *wtfB* duplicate gene is absolutely linked to the parent gene in trans, the *wtfA*+ *wtfB*+ genotype does not form. In this case, both the *wtfA*+ *wtfB−* and *wtfA− wtfB*+ genotypes independently spread in the population until the *wtfA− wtfB−* genotype is extinct (Fig. 2c).

If *wtfA*+ and *wtfB*+ are unlinked, the 2 drive genes can both spread until the driver with the highest initial frequency (i.e. the parent gene *wtfA*+) reaches fixation (Fig. 2c). The frequencies of the 2 genotypes with *wtfA*+(*wtfA*+ *wtfB*+ and *wtfA*+ *wtfB−*) when fixation of *wtfA*+ occurs varies depending on the starting allele frequencies and whether the *wtfB*+ duplicate occurs “in cis” (i.e. *wtfA*+ *wtfB*+ is the first haploid genotype with *wtfB*+) or “in trans” (*wtfA− wtfB*+ is the first haploid genotype with *wtfB*+; Fig. 2c).

### Evolution of 2 distinct *wtf* genes

**Fig. 3.**
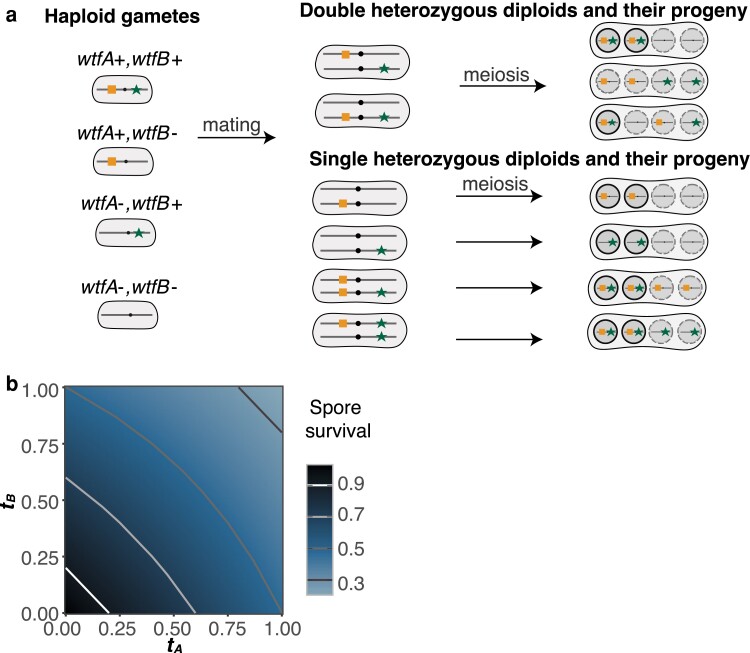
Spore survival with 2 distinct *wtf* meiotic drivers in a population. a) Cartoon of the 4 possible genotypes that carry 1 (*wtfA*+ *wtfB*− and *wtfA*− *wtfB*+), 2 (*wtfA*+ *wtfB*+), or no (*wtfA*− *wtfB*−) meiotic drivers. Haploids can mate to form diploids of a variety of genotypes, including heterozygotes which are illustrated. Drive will occur in the diploids shown as spores are susceptible to being killed by each driver they do not inherit from a heterozygote. Live spores are shown within a solid black circle whereas spores susceptible to killing by drive are shown within a dotted circle. b) The fraction of spores produced by diploids heterozygous for 2 unlinked drivers expected to survive when considering varying drive strength.

We next considered the evolution of a pair of distinct *wtf* genes (*wtfA+* and *wtfB+*) that are mutually killing. These 2 drivers could be products of a recent imperfect gene duplication, but they could also result from differential divergence of genes within a gene family in distinct lineages. Because the drivers are distinct, drive will occur in diploids heterozygous for 1 or both drivers (Fig. 3a). The *wtfA+* and *wtfB+* drivers will destroy a fraction of spores that do not inherit them from heterozygotes determined by the parameters “*t_A_”* and “*t_B_”*, respectively. When a single driver is heterozygous, the fraction of dead spores is determined by only 1 *t* parameter. When both drivers are heterozygous, the fraction of spores not inheriting both drivers that survive will be determined by both $t_{A}\;$ and $t_{B}:(1-t_{A})(1-t_{B})$. If $t_{A}\;=t_{B}=1$, only *wtfA*+ *wtfB*+ spores produced by double heterozygotes would survive. This genotype would be 25% of the total spores produced by such a diploid if the 2 genes were unlinked (Fig. 3b).

**Fig. 4.**
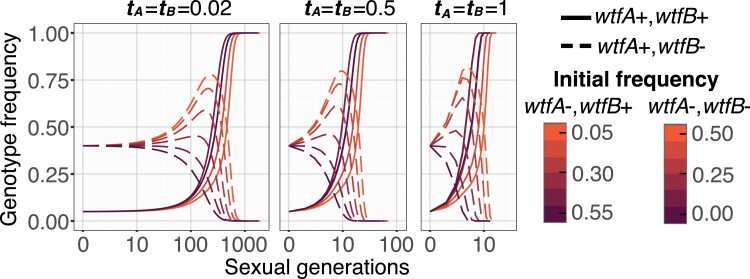
The evolution of populations with 2 drivers of equal strength in the absence of recombination. Change in driver genotype frequencies over time. The genotype frequencies of *wtfA+ wtfB*+ (solid, 0.05 initial frequency) and *wtfA+ wtfB−* (dashed, 0.40 initial frequency) with varying *wtfA*− *wtfB*− initial frequencies with 0.1 steps. The remainder of each population is comprised of the *wtfA*− *wtfB*+ genotype. The genotype *wtfA+ wtfB+* goes to fixation (See Supplementary material) when present. Strong drivers (t=1, right) spread to fixation faster than weak drivers (t=0.2, left).

We considered populations with the 2 drivers absolutely linked (r=0) in cis on the same chromosome and with drivers on distinct haplotypes absolutely linked in trans. We initially assumed that the 2 drivers were of equal strength ($t_{A}\;=t_{B}=t$). Under these conditions, we proved analytically that, if present, the *wtfA*+ *wtfB*+ genotype will spread to fixation regardless of drive strength and competing allele frequencies. (Table 2; see proof in the Supplementary material). As expected, the fixation of *wtfA*+ *wtfB*+ genotype occurred faster when the drivers were stronger (Fig. 4).

**Fig. 5.**
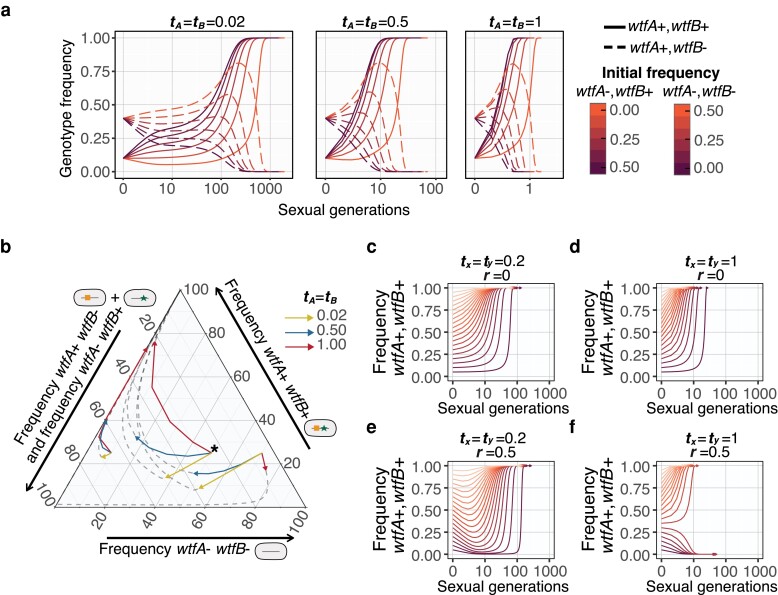
The evolution of populations with 2 drivers of equal strength in the presence of recombination. a) Changes in driver genotypes over time in the presence of recombination (*r* = 0.5). The genotype frequencies of *wtfA+ wtfB*+ (solid, 0.1 initial frequency) and *wtfA+ wtfB−* (dashed, 0.40 initial frequency) with varying *wtfA*− *wtfB*− initial frequencies with 0.1 steps. The remainder in each population is comprised of genotype *wtfA*− *wtfB*+. Strong drivers (t=1, right) spread to fixation faster than weak drivers (t=0.2, left). b) The evolution of populations that initially lack the *wtfA*− *wtfB*+ genotype. The frequency of each genotype is shown on the 3 axes. The *wtfA*− *wtfB*+ genotype can be later generated by recombination. To read the frequency of the *wtfA+ wtfB+* genotype, follow a horizontal line to the right axis. To read the frequency of the *wtfA− wtfB−* genotype, follow the diagonal down and to the left to the bottom axis. To read the combined frequency of the *wtfA+ wtfB*− and *wtfA*− *wtfB+* genotypes, follow the diagonal up and to the left to the left axis. The 2 unlinked drivers have equal strength and 3 driver strengths (indicated by the different arrow colors as shown in the key) were considered. The point marked with an asterisk (*) represents the following frequencies: *wtfA*− *wtfB*− of 0.50, *wtfA+ wtfB*+ of 0.25, and *wtfA*+ *wtfB*− plus *wtfA*− *wtfB*+ of 0.25. The arrows depict allele frequency changes over 4 generations from that starting point and the dotted lines show subsequent frequency changes. Although the frequency of the *wtfA*+ *wtfB*+ genotype can initially decline (downward arrows), that genotype eventually spreads to fixation under all conditions illustrated. c–f) Four simulated populations initially carry only 2 genotypes (*wtfA*+ *wtfB*+) and (*wtfA*− *wtfB*−). The initial frequencies for the genotype *wtfA*+ *wtfB*+ range from 0.05 to 0.95 with a 0.05 frequency step. Each simulation represents a population of 2 drivers that are absolutely linked (r=0, c and d) or unlinked (r=0.5, e and f) and have a low (t=0.2, c and e) or high transmission bias (t=1, d and f). The spread of 2 drivers is delayed by recombination as the gametes carrying 1 driver can be destroyed by the alternate driver. Strong drivers can go extinct in the presence of recombination, particularly when the starting frequency of the *wtfA*+ *wtfB*+ genotype is low (f; Table 2).

We next considered the evolution of drivers of equal strength ($t_{A}=t_{B}$) in the presence of recombination, r>0. We again found that in almost all cases, both drivers spread to fixation. As before, stronger drivers reach fixation faster (Fig. 5a–f). Interestingly, in some cases, the frequency of the double driver genotype (*wtfA*+ *wtfB*+) initially decreases prior to increasing to spread to fixation (Fig. 5b and e). This occurs when the frequency of the *wtfA− wtfB−* is relatively high and the *wtfA*+ *wtfB*+ frequency is relatively low, following the condition $x_{1}<\frac{E}{1-\bar{w}}$. In such cases, double heterozygotes are formed, and the newly created recombinant spores that inherit a single driver are thus destroyed by the opposite driver. Strikingly this effect can even lead to loss of the double driver genotype when drive is strong (t=1), no single driver genotype is present (when $x_{1}+x_{2}=1$), and the double driver genotype has a low initial frequency ($x_{1}<\frac{r}{1+r}$; Fig. 5f; Table 2).

**Fig. 6.**
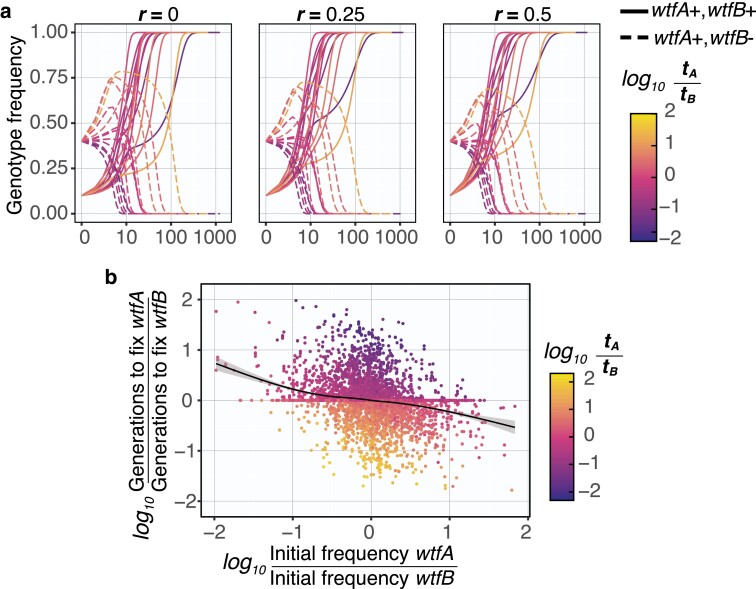
Drivers with larger transmission advantage tend to fix faster in a population. a) Simulations with varying transmission advantages $t_{A}$ and $t_{B}$ for absolutely linked (r=0), mildly linked (r=0.25) or unlinked (r=0.5)loci. The genotype frequencies of *wtfA+ wtfB*+ (solid, 0.1) and *wtfA+ wtfB−* (dashed, 0.40) with a *wtfA*− *wtfB*+ and *wtfA*− *wtfB*− initial frequencies with 0.2 and 0.3, respectively. The genotype *wtfA+ wtfB+* goes to fixation when present (see Supplementary material for mathematical proof). The frequency of the double driver genotype can decrease in the presence of recombination, but it eventually spreads to fixation when present (see Supplementary material for mathematical proof). b) Ten thousand initial populations were simulated with multiple recombination frequencies (r=0, 0.1, 0.2, 0.3, 0.4, and 0.5). The number of generations to fix a driver allele (i.e. *wtfA*) was compared to generations required to fix a second driver allele (i.e. *wtfB*). The stronger driver (larger *t*) tends to fix faster than a weaker driver, except in some cases when the weaker driver is initially more prevalent in a population. The black line is a local regression between *X* and *Y* axes. The shaded area is the standard error in the regression.

We also considered the evolution of 2 drivers of differing strength both in the presence and absence of recombination. Similar to our results with drivers of equal strength, we proved mathematically that the *wtfA*+ *wtfB*+ genotype will become fixed. Unlike the drivers of equal strength, however, there were no exceptional cases in which the *wtfA*+ *wtfB*+ genotype is not fixed when the 2 loci recombine (Table 2; see Supplementary material for mathematical proof). The *wtfA*+ *wtfB*+ fixation rate was not dramatically affected by recombination rate (Fig. 6a), and the stronger driver of the pair generally fixes faster (Fig. 6b).

### Evolution of 2 competing driving haplotypes

**Fig. 7.**
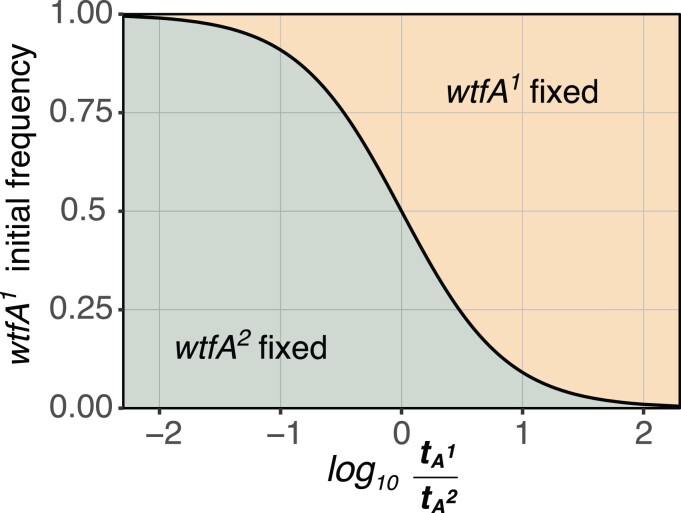
Evolution of populations with 2 allelic or absolutely linked *wtf* variants. Populations with only *wtfA^1^* and *wtfA^2^* drivers are considered to represent 2 alternate driving alleles of varying relative strengths. The plotted line (black) represents a steady state where the driver frequencies remain constant. At points above the line, the *wtfA^1^* spreads to fixation. At points below the line, the *wtfA^2^* driver spreads. The weaker driver can spread to fixation if the weaker driver starts in excess.

The *wtf* genes diverge so rapidly that different natural isolates of *S. pombe* can encode distinct drivers at a given locus (Eickbush *et al*. 2019). We therefore wanted to explore the evolution of 2 distinct *wtf* drivers found at a single locus (Eq. 3 where $x_{A^{1}}+x_{A^{2}}=1$ and r=0). The steady states in which the population remains polymorphic are unstable (Fig. 7; Table 2). We found that the stronger driver generally spreads at the expense of the weaker driver, even if it is initially present at lower frequency (Fig. 7). However, a weaker driver can drive a stronger driver to extinction if the starting frequency of the weaker driver is sufficiently high (Fig. 7; Table 2). Specifically, the weaker driver (e.g. *wtf*A^2^) will fix if:


$$x_{A^{1}}<\frac{1}{1+\frac{t_{A^{1}}}{t_{A^{2}}}}$$

where the ratio between drive strengths and the frequency of each driver determine the outcome.

## Discussion

One route to accumulate drivers within a genome could be to fix them sequentially over time. If the drivers are independent, the evolutionary dynamics of this process would be no different than single driver evolution scenarios (López Hernández *et al*. 2021). However, in some species, drivers are polymorphic (Hall and Dawe 2018; Eickbush *et al*. 2019; Vogan *et al*. 2019; Muirhead and Presgraves 2021). To better understand the evolution of such duplicates, we modeled the evolution of duplicate killer meiotic drivers.

Our goal was to better understand the dynamics of meiotic driver duplicates in general. We used the *wtf* drivers of *S. pombe* as a model. This was a strength in that the parameters describing the behavior of *wtf* drivers in the lab are known and previous modeling matched well to laboratory experimental evolution analyses (López Hernández *et al*. 2021  Wolfram Research 2021). Our study is, however, limited because we assumed an infinitely large, randomly mating population. These parameters do not describe all populations. For example, *S. pombe* grows clonally, and cells are only passively mobile, both of which disfavor outcrossing. In addition, some isolates of *S. pombe* inbreed, even in the presence of potential outcrossing partners (López Hernández *et al*. 2021). We anticipate that inbreeding would slow, but not prevent, the fixation of 2 drivers (López Hernández *et al*. 2021). Drift, however, would likely significantly diminish the number of conditions under which the 2 drivers fix with high probability as the double driver genotype could be lost to drift (López Hernández *et al*. 2021).

Our results have implications for understanding the evolution of natural drive systems, particularly poison–antidote killer meiotic drivers. Specifically, duplicates of such drive loci can be maintained or spread in a population under a broad range of conditions. This helps explain how the *wtf* genes have expanded in *Schizosaccharomyces* species. Similarly, isolates of *Podospora anserina* contain between 0 and 3 distinct *Spok* drivers (Vogan *et al*. 2019). Like the *wtf* drivers, the *Spok* drivers are encoded in a single gene, which likely facilitates their establishment after being duplicated (Vogan *et al* 2021). Partial duplication of poison–antidote drive systems in the form of antidote duplications has also been observed. For example, the first identified drive locus in the model plant *Arabidopsis thaliana* contains multiple copies of the *APOK3* gene, which encodes an antidote to an unidentified poison (Simon *et al*. 2022). Although the impact of *APOK3* duplications is unknown, such antidote duplications could potentially make a driver more efficient by ensuring extra protection for meiotic products that inherit the drive locus.

The duplication of drivers that do not use a poison–antidote mechanism may be relatively more constrained. For example, chromosome “knobs” in maize drive by preferential segregation into the egg cell during female meiosis (Sandler and Novitski 1957; Dawe *et al*. 2018). Drive of knobs is affected by chromosomal position, which likely constrains the evolution of duplicated knob sequences (Swentowsky *et al*. 2020). Knobs are also quite large, which may also limit their duplication potential. Despite these factors, multiple knobs are found on most maize chromosomes (Hufford *et al*. 2021).

Similarly, killer–target drive systems are also likely more constrained in their duplication. These drivers use a killer element to destroy the meiotic products that inherit a target locus that is found on the competing haplotype but is not found on the driving haplotype. Duplications of a killer to a location not linked in cis to the parent locus would likely be lost as the duplicate would not benefit from drive and would sometimes be destroyed by drive. However, duplications of the killer element linked in cis to the original drive locus could be favored if duplications strengthened the drive of the haplotype (Crow 1991). For example, an X chromosome–linked killer that targeted gametes inheriting the Y chromosome could duplicate on the X chromosome to enhance drive of the X. Although the mechanisms of drive are not yet known, X-linked expansions of drive genes have been observed (Kruger *et al*. 2019; Muirhead and Presgraves 2021; Vedanayagam *et al*. 2021).

Finally, this work has implications that could be considered in the design of synthetic gene drives to spread desirable traits in a population (Burt and Crisanti 2018). Single-gene poison–antidote meiotic drivers, like the *wtf* drivers, are an attractive candidate component for such synthetic gene drives. Their strong drive, small size, autonomy, and inability for the critical drive components to be uncoupled by recombination are all ideal for promoting the spread of a desired locus or chromosome in a population. Unfortunately, those same features also increase the possibility that a gene drive could spread within a genome. Such duplication could lead to less predictable control and other undesirable outcomes. As discussed above, killer–target meiotic driver systems have less duplication potential and thus may be better guides for engineering gene drives to spread desirable traits in a population, but not within genomes.

## Supplementary Material

jkae142_Supplementary_Data

## Data Availability

Original data underlying this manuscript can be accessed from the Stowers Original Data Repository at http://www.stowers.org/research/publications/libpb-2470 or by Figshare at https://doi.org/10.6084/m9.figshare.25998292.v1. Supplemental material available at G3 online.
